# Sociocultural–psychological predictors influencing parents' decision-making regarding HPV vaccination for their adolescent daughters in mainland China: An extended TPB model

**DOI:** 10.3389/fpubh.2022.1035658

**Published:** 2023-01-06

**Authors:** Lingmin Zhang, Jian Yang, Ye Cao, Wanying Kang

**Affiliations:** ^1^School of Journalism and Communication, Guangzhou University, Guangzhou, China; ^2^Archives Office, Guizhou Open University, Guiyang, China; ^3^School of Media and Communication, Shenzhen University, Shenzhen, China

**Keywords:** HPV vaccination, parental vaccination decision-making, mainland China, extended TPB model, exposure to stories, moral obligation, anticipated affective reactions

## Abstract

**Introduction:**

Enhancing human papillomavirus (HPV) vaccine uptake rates to protect women's health is an important public health issue worldwide. China has taken a series of measures in recent years to promote HPV vaccination among school-aged girls, but the vaccine uptake rate remains low. Investigating the factors influencing vaccination-related decision-making of adolescent girls' parents is key to solving the problem. This study aimed to examine the influence of sociocultural-psychological predictors, including exposure to HPV-related stories (positive/negative), affective reactions (pride/regret), injunctive norms on the Internet and perceived moral obligation, on parents' HPV vaccination-related decision-making for girls aged 13–15 years in mainland China.

**Methods:**

A cross-sectional online survey using quota sampling was conducted in February 2022. Four hundred and five valid and qualified questionnaires were obtained. Partial least squares structural equation modeling was performed by SmartPLS 3 (i) to evaluate the reliability and validity of the measurement models of 11 constructs, and (ii) to test the effect relationships of the sociocultural–psychological predictors on parents' intention to vaccinate their daughters.

**Results:**

The study findings showed that parental decision-making regarding HPV vaccination was influenced by sociocultural and psychological factors. At the level of individual psychological factors, exposure to positive stories was significantly associated with perceived vaccine effectiveness (β = 0.331, *t* = 8.448, *p* < 0.001), which strongly predicted the attitude toward vaccination (β = 0.521, *t* = 8.133, *p* < 0.001); anticipated pride had more positive influence on vaccination-related decision-making (β = 0.156, *t* = 2.176, *p* < 0.05) than anticipated regret. In terms of social influence, injunctive norms on the Internet had a significantly positive influence on vaccination intention (β = 0.127, *t* = 2.382, *p* < 0.05), similar to descriptive norms (β = 0.135, *t* = 3.358, *p* < 0.01). Perceived moral obligation at the cultural level was the strongest predictor of parental decision-making regarding HPV vaccination (β = 0.193, *t* = 2.139, *p* < 0.05).

**Discussion:**

This study is the first in mainland China to systematically examine the sociocultural-psychological predictors of parents' decision-making to vaccinate their 13–15-year-old daughters against HPV. A new extended TPB model with a sociocultural-psychological approach was developed. This model can support the investigation of factors affecting HPV vaccine uptake rates in the mainland Chinese population and similar populations and help to understand the differences in vaccination-related decision-making between Eastern and Western cultures. Furthermore, the study provided some suggestions for HPV vaccination communication campaigns targeting adolescent girls' parents.

## Introduction

As the country with the largest population, China had a crude cervical cancer incidence of 15.6 per 100,000 women in 2020 and 51,600 deaths due to cervical cancer in 2019 (1). Although the bivalent HPV vaccine was first licensed in most developed countries in 2006, it was not commercially available in mainland China until July 2016 (2, 3). Mainland China supplies three types of HPV vaccines: bivalent (Cervarix, Cecolin), quadrivalent (Gardasil), and 9-valent (Gardasil 9) vaccines, which have protective effects against high-risk HPV types 16 and 18 that are known to significantly increase the risk of cervical, vaginal, and vulvar cancer in women. Moreover, HPV vaccines are currently only available for females aged 9–45, except the 9-valent vaccine, which is only available for females aged 16–26 years. They are not approved for men in mainland China.

According to the national routine vaccine report data, the number of HPV vaccine doses administered in mainland China increased from 3.417 million in 2018 to 12.279 million in 2020 (4), but the actual coverage rate of the HPV vaccine remains low. The vaccination rate is <3% for adolescent girls and <6% for the whole population (5).

World Health Organization (WHO) suggested that 90% of girls should be fully vaccinated against HPV by 15 years of age by 2030 (6). In recent years, the Chinese government has attached great importance to the promotion of HPV vaccination and recently implemented the policy of free vaccination with the bivalent vaccine for middle school-aged girls in several pilot cities (7). Despite these efforts, China is still a long way from achieving a high HPV vaccine uptake rate in eligible adolescent girls because of inadequate availability of vaccine and various barriers to vaccine acceptance (8). Parents are usually the decision-makers regarding HPV vaccination for their 13–15-year-old daughters. Thus, Understanding predictors of parental decision-making for adolescent daughters' HPV vaccination can inform strategies to increase vaccination uptake in mainland China. Previous studies have investigated the vaccine hesitancy of mainland Chinese parents, and found that the level of knowledge, the daughters' age, awareness of HPV infection risks, vaccine safety and efficacy, peer influence, and costs were significant influencing factors (9–13). However, most studies have been descriptive, and have lacked a systematic explanation of the factors influencing parents' HPV vaccination intention for their adolescent daughters.

### The extended theory of planned behavior model

The theory of planned behavior (TPB) is a representative theory about the relationship between attitude and behavior in the field of social psychology. It posits that attitude, subjective norms, and perceived behavioral control (PBC) are the three mutually influencing factors that affect a person's behavioral intention, which is in turn the factor that most directly influences actual behavior. Attitude toward a behavior refers to an individual's overall evaluation of a behavior. Subjective norms are described as an individual's perceived social expectations for a behavior. PBC is defined as an individual's perceived confidence in performing it, and it is commonly measured as self-efficacy in performing a behavior (14).

Since Ajzen suggested that the TPB model is open to expansion (14), many researchers have proposed the additional new predictors to improve the explanatory power of the original model (15–17). Compared with other theory models of health behavior, the extended TPB model has the advantage of incorporating the influence factors at the social level in addition to psychological aspects, which has been widely used as a theoretical framework to examine vaccination intention and behavior in different populations and contexts around the world (18–25).

A large number of studies in Western countries have confirmed the explanatory power of attitude, subjective norms, and PBC on parental HPV vaccination intention for their children. Other determinants, including media use, perceptions of HPV infection and vaccination risks, descriptive norms, and anticipated regret have also been included in extended TPB models to explore their impact on behavioral intention (26–33). There have been many studies of vaccination hesitancy in Western countries based on the TPB model, but they have not fully explained parental HPV-vaccination-related decision-making in the social and cultural contexts of mainland China.

China has a different social culture from Western societies, as it emphasizes collectivism and child-centered family traditions, greater obedience to authority, and a higher tolerance for uncertainty (34). This leads to corresponding psychological characteristics. Therefore, Chinese parental HPV vaccination decision-making is influenced by both individual-level psychological factors and the sociocultural context at the super-individual level. It is necessary to explore sociocultural psychological factors adapted to the local situation to predict Chinese parents' intention to vaccinate their 13–15-year-old daughters.

### Influence of parents' stories exposure on attitude toward vaccination

Attitude is the strongest predictor of vaccination intention in the TPB model. The established TPB model emphasizes the cognitive antecedents of vaccination attitude, e.g., risk perception, but ignores the factors affecting the cognitive antecedents. Recent studies have shown that exposure to messages from different channels can shape people's knowledge and attitudes toward HPV vaccines (35–39). HPV vaccination-related stories in the messages may be an effective tool in influencing people to vaccinate or not (40). Among the HPV vaccination-related information that parents are exposed to through various channels, there are generally two types of stories: positive and negative. Positive stories convey that HPV vaccination can be beneficial to women's health; negative stories emphasize the different degrees of personal safety accidents caused by HPV vaccination. In one study, compared with participants exposed to positive messages, those who were exposed to negative messages about HPV vaccination perceived the vaccine as less safe, took more negative attitudes toward vaccination, and expressed less willingness to vaccinate (41). However, how the exposure to positive and negative stories in social media have shaped mainland Chinese parents' vaccination risk perception and attitudes toward HPV vaccination is poorly understood.

### Influence of anticipated emotional reactions on vaccination intention

The variable of attitude in TPB model only emphasizes the cognitive component, and ignores the emotional component that influences health behavioral intention and decision-making (42–45). Anticipated emotional reactions are defined as people's expectations of the affective responses they are likely to experience after performing a particular behavior, and are centered around self-conscious emotions such as pride, regret, and guilty (44, 46). Some previous studies have investigated the effect of anticipated regret of not being vaccinated, and have shown that it has a significant effect on HPV vaccination intention (29, 46–50). However, the role of the positive affective reaction of anticipated pride if vaccinated has been less frequently discussed. These two factors at the psychological level have not been evaluated in studies of mainland Chinese parents' HPV vaccination decision-making.

### The effects of descriptive and injunctive norms on vaccination intention

The variable of subjective norms in the TPB model emphasizes the social and cultural factors affecting individuals' health-related decision-making. It includes two aspects: descriptive norms (copying others' behavior) and injunctive norms (behaving as others expect). Descriptive norms has been proven to be a significant influence factor on vaccination attention (18, 51), while injunctive norms (behaving as others expect) has a weak effect. Moreover, most studies have only investigated one of these aspects in the survey, and few have simultaneously validated and compared the impacts of both aspects. In the Chinese society centered with collectivism, the demands and expectations of social groups are an important source of influence in shaping people's behavior. As the development of the Internet technology expands the scope of primary groups, it is necessary to discuss the impact of the injunctive norms of social groups on the Internet on parental vaccination intention in this study.

### Perceived moral obligation predicting vaccination intention

Ajzen suggested that moral norms, along with attitude, subjective norms, and PBC, directly affect intentions (14). Several studies have paid attention to parents' moral obligation to vaccinate their children (52–54), but this type of research is lacking in the context of mainland Chinese parents in the collectivistic and child-centered culture. In Chinese culture, the whole society creates a family culture based on close parent-child relationships, parents are responsible for nurturing and preparing their children to achieve socialization goals (55, 56). Currently, Chinese parents generally present a consensus on ethical responsibility: everything is for the child and the best for the child. With improvements in the socioeconomic status of Chinese families and the popularity of scientific parenting ideology, Chinese parents are willing to adopt scientific methods to manage their children's health and strive to improve their own “quality of care” (57). It is important to include the cultural factor of moral obligation in the model to understand the Chinese phenomenon.

### The current study

Based on the local situation in mainland China, this study aimed to assess the impact of sociocultural-psychological predictors affecting mainland Chinese parents' decision-making regarding HPV vaccination for their adolescent daughters. We aimed to test the relationships between the influence of exposure to positive or negative stories and perceived vaccination risk, including vaccine effectiveness and vaccine side-effects, and attitude toward vaccination. Further, we aimed to explore the effects of three levels of psycho-emotional, social norm, and cultural influences, namely, anticipated emotional reactions (anticipated regret and pride), injunctive norms on the Internet, and perceived moral obligation, on parents' vaccination intention for their daughters. We proposed following hypothesis:

*H1: Exposure to positive stories has a positive influence on perceived vaccine effectiveness*.
*H2: Exposure to negative stories has a positive influence on perceived vaccine side-effects*.
*H3: Perceived vaccine effectiveness has a positive influence on attitude toward vaccination*.
*H4: Perceived vaccine side-effects has a negative influence on attitude toward vaccination*.
*H5: Perceived infection risk has a positive influence on attitude toward vaccination*.
*H6: Attitude toward vaccination has a positive influence on vaccination intention*.
*H7: Anticipated regret has a positive influence on vaccination intention*.
*H8: Anticipated pride has a positive influence on vaccination intention*.
*H9: Descriptive norms has a positive influence on vaccination intention*.
*H10: Injunctive norms on the Internet has a positive influence on vaccination intention*.
*H11: Perceived moral obligation has a positive influence on vaccination intention*.
*H12: Self-efficacy for vaccination has a positive influence on vaccination intention*.

**Figure 1:**
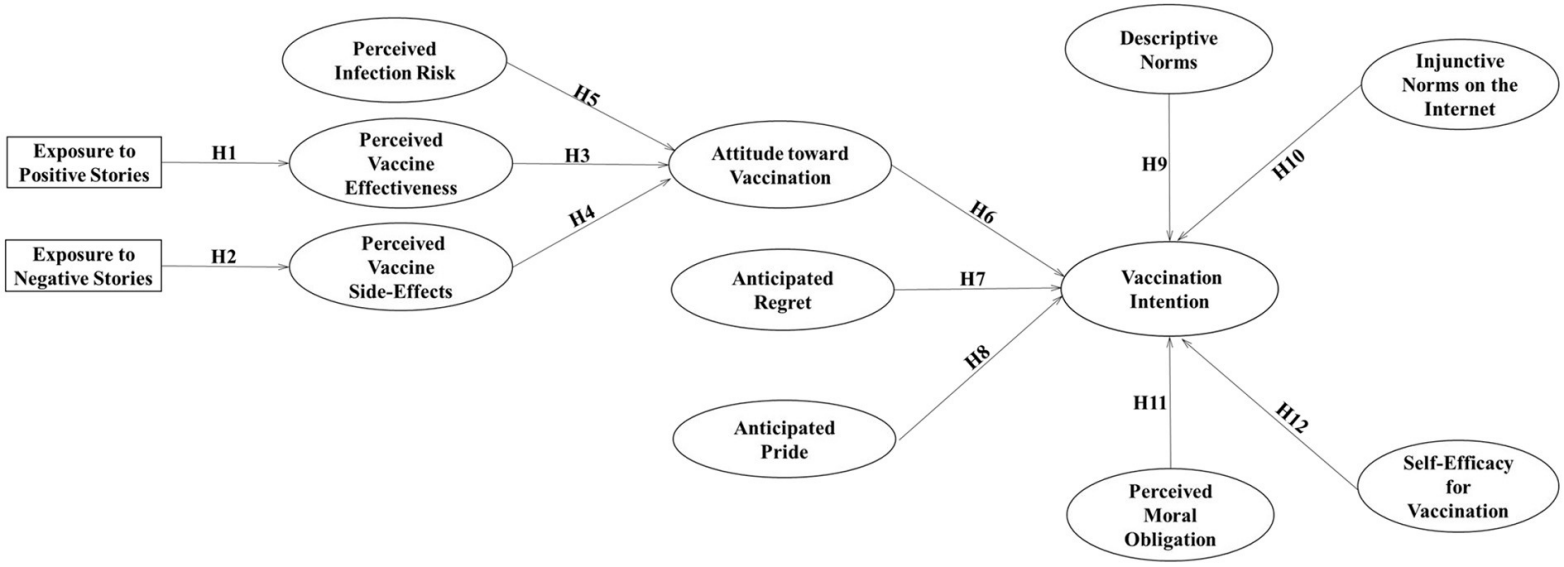
Extended theoretical framework of the theory of planned behavior model.

Based on Hypotheses 1–12 as proposed above, Figure 1 presents the theoretical framework of this study.

## Methods

### Data and sample

In February 2022, a cross-sectional anonymous online survey of mainland Chinese parents of adolescent girls aged 13–15 years was conducted on a professional online research platform (Survey Plus). Quota sampling based on the educational level and vaccination status (yes/no) was used to ensure that the samples adequately represented the specific demographic characteristics of general Chinese parents. At the beginning of the questionnaire, the participants were given an explanation of the study purpose and procedures. Participation in the study was voluntary, and the participants could withdraw at any time without any consequence.

To obtain qualified data, we included several quality control procedures in the questionnaire system, including an Internet Protocol duplicate check avoiding multiple answers by one participant, an answering time of at least 15 min, an attention test preventing participants from answering indiscreetly, and a logical relationship check to identify unqualified questionnaires with contradictory answers. After rigorous data check and cleaning, 405 valid questionnaire responses were obtained. Of the total survey respondents, 30.4% were male and 69.6% were female. This sex ratio is reasonable as in Chinese families, mothers are often responsible for the daily lives and health of their children. The average age of the respondents was 39.1 years (standard deviation = 3.36). Table 1 shows the demographic characteristics of the total sample, including sex, educational level, region, average monthly household income, and daughter's HPV vaccination status.

**Table 1:** Sociodemographic data of the respondents.

	**Frequency**	**Percentage**
**Sex**		
Male	123	30.4%
Female	282	69.6%
**Educational level**		
Secondary school or lower	99	24.4%
High school or equivalent	104	25.7%
College or equivalent	185	45.7%
Postgraduate	17	4.2%
**Region**		
Urban	273	67.4%
Rural	132	32.6%
**Average monthly household income**		
RMB 5,000 and below	65	16.0%
RMB 5,001–10,000	102	25.2%
RMB 10,001–15,000	101	24.9%
RMB 15,001–20,000	76	18.8%
RMB 20,001–30,000	36	8.9%
RMB 30,001 or above	25	6.2%
**Daughter's HPV vaccination status**		
Yes	155	38.3%
No	250	61.7%

### Measures

#### Exposure to positive stories

Adapted from previous research (40), the variable of exposure to positive stories on HPV vaccination-related information was measured using a multiple-choice question with seven options: Have you heard the stories that HPV vaccination can prevent (1) genital warts; (2) HPV infection; (3) an abnormal Pap smear; (4) an HPV-related health problem; (5) death from an HPV-related cancer; (6) another HPV-related health problem; or (7) none of these? We assigned a different score to each option according to its positiveness: one point for the first, second, and sixth options; two points for the third option; three points for the fourth option; four points for the fifth option; and zero points for the seventh option. Finally, we used the summed total score of each parent as an index to measure the positiveness of the information to which each parent had been exposed.

#### Exposure to negative stories

The parents indicated the content of negative stories describing people being harmed by HPV vaccine using a multiple-choice question with five response options: Have you heard the stories that HPV vaccine (1) had mild side-effects; (2) had serious temporary harms; (3) had serious long-lasting harms; (4) caused death; or (5) had other harms? We assigned a different score to each option according to its negativity: one point for the first and fourth options, two points for the second option, three points for the third option, and zero points for the fifth option. Finally, we used a summed total score for each parent as an index to measure the negativity of the HPV vaccination-related information to which each parent had been exposed.

#### Seven-point Likert Scales for constructs

In addition to the above two variables, 11 other constructs were measured.

Based on the findings in the literature, localized modifications were made to the scales, and the scales were translated into Chinese. Seven-point Likert scales were used. Items below the factor loading value threshold were eliminated. The details of the scales for the study constructs were provided in the Appendix.

### Data analysis

Partial least squares structural equation modeling (PLS-SEM) has been demonstrated to be effective for verifying complex influence relationships in path models with latent variables (58). Our research model comprised 12 direct influence hypotheses, and the study aimed to establish an expanded TPB model to explore the influencing factors of and mechanism underlying parents' decision-making regarding HPV vaccination for their 13–15-year-old daughters in the sociocultural context of mainland China. Therefore, the PLS-SEM was suitable for this study. The tool of SmartPLS 3 was used for model analysis in the research. The whole data analysis consisted of two parts: measurement model evaluation and structure model evaluation (59–61). Cronbach's α and composite reliability were used to test the reliability of the 11 constructs, and the average variance extracted to test their convergent validity. The Fronell–Larcker criterion and heterotrait–monotrait ratios of correlations were used to check the discriminant validity of measurement models. The part of structure model evaluation verified four sets of cause–effect relationships: (1) between exposure to positive stories and perceived vaccine effectiveness; (2) between exposure to negative stories and perceived vaccine side-effects; (3) between perceived infection risk, perceived vaccine effectiveness, perceived vaccine side-effects, and attitude toward vaccination; and (4) between attitude toward vaccination, subjective norms (descriptive norms and injunctive norms on the Internet), anticipated affective reactions (anticipated regret and anticipated pride), perceived moral obligation, self-efficacy for vaccination, and vaccination intention. The determination coefficient (*R*^2^) and the predictive correlation (*Q*^2^) were used to indicate the qualification of the four structure models. Variance inflation factors were adopted to examine the multicollinearity issue.

## Results

### Measurement model evaluation

The first part shows the results of measurement model evaluation that indicate the rationality of the measurement models through reliability, convergent validity, and discriminant validity.

Table 2 presents the results of reliability and convergent validity. The values of Cronbach's α were between 0.815 and 0.977 (cut-off > 0.7), and the composite reliability values ranged from 0.880 to 0.983 (cut-off > 0.7), showing that the internal consistency and reliability of the measurement model were good (62, 63).

**Table 2:** Results of reliability and convergent validity.

**Constructs**	**Measurement items**	**Factor loading**	**Cronbach's alpha**	**Composite reliability**	**Average variance extracted**
Perceived infection risk (PIR)	PIR1 PIR2 PIR3	0.857 0.837 0.834	0.815	0.880	0.710
Perceived vaccine effectiveness (PVE)	PVE1 PVE2 PVE3	0.949 0.958 0.926	0.939	0.961	0.891
Perceived vaccine side-effects (PVSE)	PVSE1 PVSE2 PVSE3	0.925 0.960 0.936	0.935	0.958	0.885
Attitude toward vaccination (AV)	AV1 AV2 AV3 AV4	0.944 0.938 0.948 0.942	0.958	0.970	0.889
Anticipated regret (AR)	AR1 AR2 AR3 AR4	0.964 0.971 0.972 0.961	0.977	0.983	0.936
Anticipated pride (AP)	AP1 AP2 AP3	0.931 0.943 0.946	0.934	0.958	0.884
Descriptive norms (DN)	DN1 DN2 DN3 DN4 DN5 DN6	0.864 0.868 0.940 0.949 0.943 0.933	0.962	0.969	0.841
Injunctive norms on the Internet (INI)	INI1 INI2 INI3	0.958 0.957 0.959	0.955	0.971	0.918
Perceived moral obligation (PMO)	PMO1 PMO2 PMO3 PMO4	0.932 0.929 0.949 0.820	0.929	0.950	0.826
Self-efficacy for vaccination (SEV)	SEV1 SEV2 SEV3	0.908 0.851 0.940	0.884	0.928	0.811
Vaccination intention (VI)	VI1 VI2 VI3	0.964 0.972 0.972	0.968	0.979	0.940

All factor loadings ranged from 0.820 to 0.972, and the average variance extracted values were between 0.710 and 0.940, which are higher than 0.5 (59, 63). This indicates that the convergent validity of the measurement model was good.

The results of the Fornell–Larcker criterion and heterotrait–monotrait ratio of correlation analyses revealed that the measurement model had good discriminant validity among these constructs (Table 3). In Table 3, the square roots of average variances extracted on each construct are greater than the Pearson's correlation coefficients between the constructs (63). All heterotrait–monotrait ratios of correlations ranged from 0.026 to 0.776 (cut-off < 0.85) (64).

**Table 3:** Results of Fornell–Larcker criterion and heterotrait–monotrait ratio of correlation analyses.

	**PIR**	**PVE**	**PVSE**	**AV**	**AR**	**AP**	**DN**	**INI**	**PMO**	**SEV**	**VI**
Perceived infection risk (PIR)	**0.843**	0.284	0.080	0.252	0.226	0.220	0.272	0.254	0.337	0.262	0.200
Perceived vaccine effectiveness (PVE)	0.272	**0.944**	0.229	0.604	0.128	0.580	0.321	0.482	0.560	0.624	0.460
Perceived vaccine side-effects (PVSE)	0.037	−0.215	**0.941**	0.225	0.026	0.176	0.091	0.178	0.163	0.203	0.099
Attitude toward vaccination (AV)	0.247	0.574	−0.213	**0.943**	0.325	0.776	0.489	0.648	0.761	0.707	0.652
Anticipated regret (AR)	−0.236	−0.123	−0.001	−0.314	**0.967**	0.357	0.238	0.291	0.375	0.266	0.201
Anticipated pride (AP)	0.221	0.544	−0.165	0.735	−0.34	**0.94**	0.471	0.630	0.737	0.701	0.643
Descriptive norms (DN)	0.255	0.305	−0.087	0.470	−0.229	0.446	**0.917**	0.590	0.510	0.519	0.516
Injunctive norms on the Internet (INI)	0.252	0.456	−0.168	0.620	−0.281	0.595	0.563	**0.958**	0.669	0.612	0.604
Perceived moral obligation (PMO)	0.321	0.527	−0.152	0.722	−0.355	0.69	0.482	0.631	**0.909**	0.761	0.674
Self-efficacy for vaccination (SEV)	0.240	0.572	−0.182	0.657	−0.247	0.643	0.567	0.567	0.698	**0.901**	0.660
Vaccination intention (VI)	0.206	0.439	−0.094	0.628	−0.196	0.614	0.582	0.582	0.641	0.617	**0.969**

Numbers in bold font are the square roots of average variance extracted.

### Structure model evaluation

The determination coefficient (*R*^2^) was used to confirm the effects of external variables on internal dependent variables (65), and the predictive correlation (*Q*^2^) value was used to indicate whether the structural model could accurately predict the data (59). Table 4 shows that the *R*^2^ values ranged from 0.110 to 0.542 (cut-off > 0.1), reflecting that the external variables in the model had a notable impact on the internal dependent variables (58). All *Q*^2^ values are above 0, indicating that the structural model in this study was highly capable of predicting the data (64, 66).

**Table 4:** Results of *R*^2^ and *Q*^2^ analyses.

	***R* ^2^**	***Q* ^2^**
Perceived vaccine effectiveness	0.110	0.097
Perceived vaccine side-effects	0.232	0.203
Attitude toward vaccination	0.348	0.303
Vaccination intention	0.542	0.501

**Figure 2:**
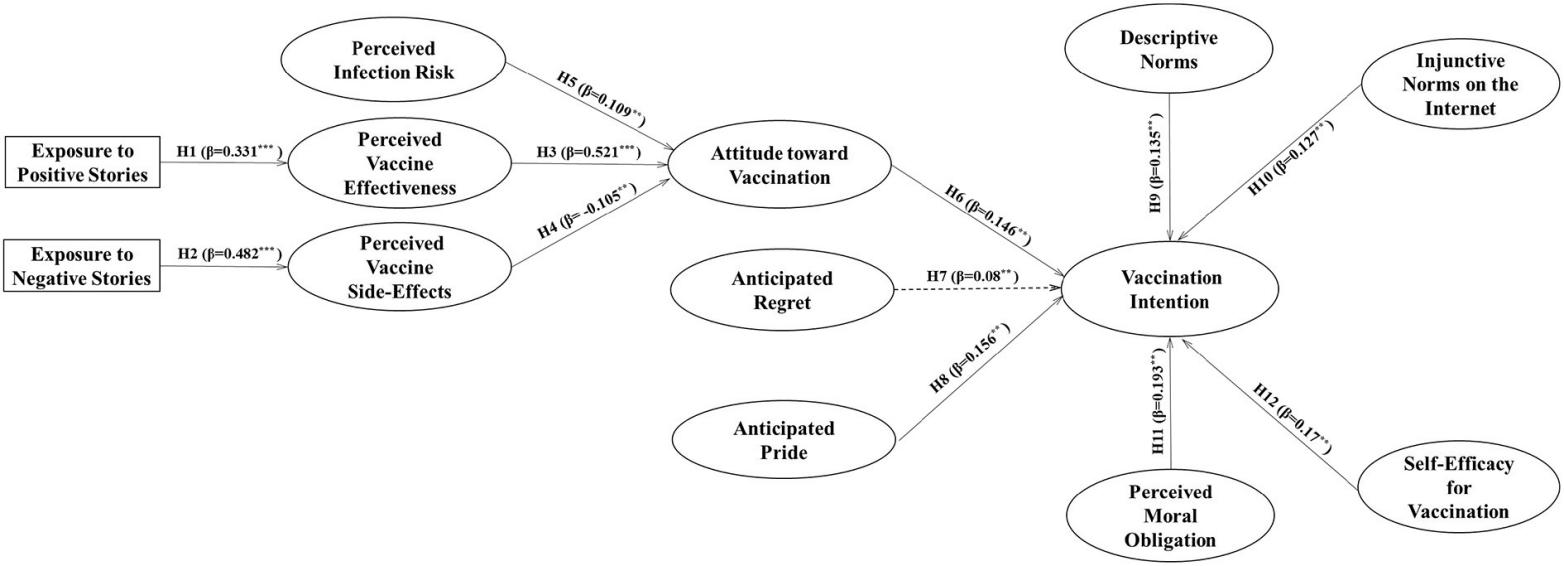
Results of hypothesis testing in the structural equation model. ***p < 0.001; **p < 0.01.

The bootstrapping resampling method (5,000 resamples) was used to test the statistical significance of the variables. The results are shown in Table 5, Figure 2. Exposure to positive stories was significantly associated with perceived vaccine effectiveness (β = 0.331, *t* = 8.448, *p* < 0.001), while exposure to negative stories was significantly associated with perceived vaccine side-effects (β = 0.482, *t* = 11.119, *p* < 0.001). This confirmed our H1 and H2. The three variables of perceived vaccine effectiveness, perceived vaccine side-effects, and perceived infection risk were all significantly related to attitude toward vaccination. Among them, perceived vaccine effectiveness and perceived infection risk had positive effects on the attitude toward vaccination, with path coefficients of 0.521 (*t* = 8.133, *p* < 0.001) and 0.109 (*t* = 2.456, *p* < 0.05), respectively, while perceived vaccine side-effects had a negative effect on the attitude toward vaccination (β = −0.105, *t* = 2.384, *p* < 0.05). Thus, our H3, H4, and H5 were supported. Regarding the cause–effect relationships between the seven independent variables and the dependent variable of HPV vaccination intention, except for the variable of anticipated regret, all of the other six variables showed significant effects on HPV vaccination intention. The path coefficients of attitude toward vaccination and anticipated pride to vaccination intention were 0.146 (*t* = 2.232, *p* < 0.05) and 0.156 (*t* = 2.176, *p* < 0.05) respectively, supporting H6 and H8; the path coefficients of descriptive norms and injunctive norms on the Internet to vaccination intention were 0.135 (*t* = 3.358, *p* < 0.01) and 0.127 (*t* = 2.382, *p* < 0.05), supporting H9 and H10. H11 and H12 are also supported, as the path coefficients of perceived moral obligation and self-efficacy for vaccination to HPV vaccination intention were 0.193 (*t* = 2.139, *p* < 0.05) and 0.170 (*t* = 2.450, *p* < 0.05), respectively. Moreover, multicollinearity was mainly detected by the variance inflation factor (67). Appendix Table 1 shows that all variance inflation factors ranged from 1.000 to 2.922, indicating no multicollinearity issue (68).

**Table 5:** Results of hypothesis verification.

**Hypothesis and paths**	**β-values**	***t*-values**	***p*-values**	**Variance inflation factor**	**Result**
*H1: Exposure to positive stories*→*Perceived vaccine effectiveness*					
	0.331	8.448	0.000	1.000	Accept
*H2: Exposure to negative stories*→*Perceived vaccine side-effects*					
	0.482	11.119	0.000	1.000	Accept
*H3: Perceived vaccine effectiveness*→*Attitude toward vaccination*					
	0.521	8.133	0.000	1.143	Accept
*H4: Perceived vaccine side-effects*→*Attitude toward vaccination*					
	−0.105	2.384	0.017	1.059	Accept
*H5: Perceived infection risk*→*Attitude toward vaccination*					
	0.109	2.456	0.013	1.091	Accept
*H6: Attitude toward vaccination*→*Vaccination intention*					
	0.146	2.232	0.012	2.898	Accept
*H7: Anticipated regret*→*Vaccination intention*					
	0.080	2.161	0.031	1.176	Reject
*H8: Anticipated pride*→*Vaccination intention*					
	0.156	2.176	0.030	2.646	Accept
*H9: Descriptive norms*→*Vaccination intention*					
	0.135	3.358	0.001	1.566	Accept
*H10: Injunctive norms on the Internet*→*Vaccination intention*					
	0.127	2.382	0.017	2.133	Accept
*H11: Perceived moral obligation*→*Vaccination intention*					
	0.193	2.139	0.032	2.922	Accept
*H12: Self-efficacy for vaccination*→*Vaccination intention*					
	0.170	2.450	0.014	2.321	Accept

## Discussion

Consistent with previous studies (26, 29–31, 69), our findings support the explanatory power of the traditional TPB model. The three core factors of attitude, subjective norms, and PBC all were found to positively affect mainland Chinese parents' decision-making regarding HPV vaccination for their daughters. Mainland Chinese parental decision-making regarding vaccination for children is not entirely a rational trade-off between pros and cons, but is complicated by many social, cultural and psychological factors. A new extended TPB model with a sociocultural-psychological approach was developed to examine the determinants of parental decision-making regarding HPV vaccination in mainland China. This model can support the investigation of factors affecting HPV vaccine uptake rates in the mainland Chinese population and similar populations and help to understand the differences in vaccination-related decision-making between Eastern and Western cultures.

Both perceived infection risk and perceived risks associated with vaccination (vaccine effectiveness and vaccine side-effects) are important factors affecting attitude toward vaccination (70). This study was conducted to answer a fundamental question: Why do people hold such cognitive beliefs? Our findings further reveal that the type of HPV vaccination-related stories/information (negative vs. positive) had a substantial impact on parents' perception of the risk associated with vaccination. When the parents were exposed to more positive stories about HPV vaccination, their perceptions of vaccine effectiveness were stronger (Hypothesis 1); however, the perceived vaccine side-effects were stronger when the parents were exposed to more negative stories (Hypothesis 2). In this study, compared with their perceptions of HPV infection risk and vaccine side-effects, parents' perceptions of vaccine effectiveness had a stronger impact on their attitudes toward vaccination. Therefore, the effects of exposure to negative stories about HPV vaccination are of little concern.

Studies on parental decision-making regarding vaccination in Western countries have shown that anticipated negative affective reactions, such as anticipated regret, anticipated worry, and anticipated anxiety, were significantly associated with increased parental intention to have their children vaccinated (29, 50, 71, 72). But in our mainland Chinese sample, anticipated regret if not vaccinated had no significant effect on parents' vaccination intention (Hypothesis 7), while anticipated pride if vaccinated positively predicted parents' vaccination intention for their daughters (Hypothesis 8).

Some studies have shown that anticipated regret is a key factor affecting parents' decision to vaccinate their daughters in the case of mandatory vaccination or during highly contagious and devastating pandemics (69, 73). HPV vaccines are not included in the scope of compulsory vaccination in mainland China, and Chinese parents, like parents in other countries, believe that their daughters are too young to be infected with HPV and that even if they are infected, they may not necessarily develop cervical cancer (74, 75). This explains why the negative affective reaction of anticipated regret was not found to have a significant impact on parental decisions regarding HPV vaccination for their daughters in our study, as parents considered vaccination neither necessary nor urgent. In addition, negative affective responses tend to be effective for those who have no emotional involvement in health behaviors, as health behaviors are more likely to attract their attention and interest (76). However, the Chinese parents in our survey were mostly mothers, who are highly emotionally involved in the physical health of their daughters, especially in terms of female health issues. The third explanation is that anticipated regret over a future negative outcome that may not necessarily occur may not be enough to enhance parents' willingness to vaccinate their daughters. Instead, the anticipated pride a person may feel immediately after engaging in a health behavior may be a more significant factor influencing decisions related to vaccination (48). As a participant said, “The government does not demand parents to vaccinate daughters against HPV. It is more likely to be recommended by professional authorities or the social media. In other words, if you don't vaccinate your children, you won't be criticized, but if you do, you will be praised.”

In addition to psychological factors, social rules and parenting culture are important factors affecting Chinese parents' decision-making regarding HPV vaccination for their daughters. The result of our study revealed the positive influence of descriptive norms on vaccination intention (Hypothesis 9), which is consistent with those of previous studies (29, 34, 72, 77). Moreover, this study has showed the significant impact of injunctive norms on the Internet (netizens' requirements and expectations of decision-makers) on parents' decision-making regarding vaccinating their daughters (Hypothesis 10).

Since the 1980's, China has entered the era of market economy, and material life has become extremely rich. The quality of life of children who grew up after the 1990's is much better than that of their parents. Because parents and grandparents place all of their expectations on these children and take care of them meticulously, many of the children are called “little emperors” by the media (78). As prenatal and postnatal care policy has become an important part of China's population development strategy, parenting is no longer a private matter restricted within the family; rather, it has become a public issue that concerns the whole society and is dictated by a set of scientific methods and practical guidelines. Therefore, it is parents' responsibility to fulfill the social requirements and expectations of parenting. Under the influence of such social rules, the significant relationship between the variable of injunctive norms and parental decision-making regarding HPV vaccination is understandable. Additionally, the Internet is increasingly permeating all aspects of people's daily lives, and many life scenarios are built on the Internet. Therefore, in addition to the traditional primary groups emphasized in sociology, such as family and friends, the influence of experts, media, and netizens on the Internet is increasingly highlighted.

The effectiveness of injunctive social norms depends on the Chinese culture of “Wangzichenglong.” Every Chinese parent has a strong sense of ethical responsibility, and the normative value of “everything is for the child” has been deeply embedded in their daily parenting practices. This study also has confirmed a positive relationship between the variable of perceived moral obligation and vaccination decision-making, with this variable showing the strongest impact coefficient among all of the influencing factors (Hypothesis 11). The salient effects of injunctive norms and perceived moral obligation also help us to understand the role of anticipated pride. When parents take the initiative to complete the non-mandatory HPV vaccination for their daughters, they meet the high requirements and expectations of society regarding parenting and fulfill the parental responsibility of “the best for the child.” This elicits a sense of pride— “I am a competent parent”—indicating that this positive affective reaction strengthens the parents' intention to vaccinate their daughters.

## Limitations

First, the findings of our study should be verified in different contexts, such as different socioeconomic classes and regions. Although parents with low educational levels and from rural regions were included in this survey, the representation of these populations was insufficient as the majority of our sample belonged to the middle and higher socioeconomic classes. Parents of different socioeconomic classes tend to have different parenting views (57); therefore, future research should include more parents of lower socioeconomic classes, particularly migrant workers and low-income groups, to further verify the applicability of this extended TPB model to the general population.

Second, the subject of this study was the parents' vaccination decision-making only for girls aged 13–15 years old. Decision-making regarding the vaccination of boys was not included. There is no HPV vaccine available for men in mainland China, and most members of the public believe that HPV vaccination is exclusively for women. However, we should be aware that it is a global trend to include men in HPV vaccination programs, which is beneficial to eliminate the adverse effects of HPV infection on human health worldwide. Therefore, future research needs to pay attention to the uniqueness of the vaccination decision-making behavior of parents of school-aged boys and the establishment of new explanatory models of factors affecting this process, and provide effective recommendations for future policy-making on HPV vaccination for the all age-qualified populations including men.

Third, social, cultural, and psychological factors are known to be intertwined, and thus, their effects on parental decision-making regarding HPV vaccination are interdependent. Consistently, this study also found correlations between injunctive norms on the Internet, perceived moral obligation, and anticipated pride. Therefore, future research should further explore the specific relationships between these three variables to enrich the expanded TPB model.

Finally, this study is the first to explore the factors influencing parental vaccination intention, but the findings are still insufficient to help improve the HPV vaccine uptake rate in the target population in mainland China, as there is always a gap between vaccination intention and vaccine uptake (18, 29). Therefore, we suggest that future studies incorporate the vaccine uptake rate into the extended TPB model and conduct continuous sample surveys to identify the factors influencing vaccine uptake and clarify the relationship between vaccination intention and vaccine uptake.

## Conclusions and public health implications

The question of how to increase the willingness of parents to vaccinate their school-aged daughters against HPV is an important issue in mainland China. In this study, we found the following answers: (1) parents in mainland China are less exposed to positive stories about HPV vaccines, and their awareness of HPV vaccine efficacy is insufficient, resulting in an insufficiently strong desire to vaccinate their adolescent daughters; (2) parents' sense of ethical responsibility to be good parents and the anticipated pride brought about by their daughters' HPV vaccination has not been effectively encouraged; (3) parents have not felt the expectations and requirements of society, including information and views provided on the Internet, to adopt vaccination behaviors; and (4) the current supply of HPV vaccines in mainland China is not sufficient and the cost is relatively high, which together, discourage parents in mainland China from behavioral intentions of HPV vaccination for their daughters.

This study is the first in mainland China to systematically examine the sociocultural–psychological predictors of parents' decision-making to vaccinate their 13–15-year-old daughters against HPV. An extended TPB model incorporating the variables of exposure to vaccination-related stories (positive/negative), anticipated affective reactions (regret/pride) injunctive norms on the Internet and perceived moral obligation was developed. The findings showed that the cultural predictor of perceived moral obligation had the strongest impact on parents' vaccination intention for their daughters. At the social level, this study newly discovered the positive relationship between the injunctive norms on the Internet and parental vaccination intention. Further, we found that the exposure to positive stories about HPV vaccination positively affected parents' perceived vaccine effectiveness, which in turn had a strong positive impact on their attitudes toward vaccination for their daughters. However, we found a weak impact of exposure to negative stories on parents' attitudes. Importantly, the study demonstrated that the impact of the positive affective response (anticipated pride) on parents' decision-making was stronger than the impact of their attitudes, whereas the negative affective response (anticipated regret) has no significant impact on vaccination intention. Self-efficacy for vaccination also was found to be a powerful factor influencing parental decision-making regarding vaccination.

In a word, the research findings showed that the influence of the sociocultural-psychological predictors on parental decision-making regarding HPV vaccination in the context of China emphasizing collectivism and family culture of “children first.” The study contributed to the research of parents' HPV decision-making considering comprehensively local social culture and individual's affective emotions. Based on these findings, the following suggestions are proposed for future public health campaigns on HPV vaccination.

Communication campaigns for HPV vaccination in mainland China should specifically target the parents of 13–15-year-old girls and actively disseminate positive yet scientific HPV vaccination-related stories mainly on social media or other channels. This could help to enhance parents' understanding of the effectiveness of HPV vaccines in preventing HPV infection and cervical cancer. Further, we found that although exposure to negative stories tended to strengthen parents' perception of vaccine side-effects, they did not have a strong negative impact on attitude toward vaccination; thus, scientific communicators need not worry too much about negative stories on social media.

Furthermore, perceived moral obligation and anticipated pride were found to be the two main factors affecting parents' decision-making regarding vaccination for their daughters. Thus, we suggest that communication contents be designed around these two aspects. On the one hand, communications could highlight the vaccination of daughters against HPV as the responsibility of competent parents; on the other hand, communications could emphasize the sense of pride that daughters' vaccination could elicit in the parents, which would help to further stimulate parents' willingness to vaccinate their daughters.

In terms of social influence, in addition to paying attention to the influence of traditional primary groups, such as relatives and friends, the influence of netizens, experts on the Internet, and social media should also be considered (79). In addition to using social media as a channel for disseminating positive stories to create a favorable environment, health communication campaigns could be implemented to strengthen the influence of online groups and online communities in promoting parents' vaccination intention.

Currently, the shortage of HPV vaccine supply and the high cost of these vaccines in mainland China are barriers to parental decision-making regarding vaccination. Therefore, public health policymakers should strive to eliminate these barriers by increasing the supply of vaccines and fully implementing free vaccination for school-aged girls to improve the self-efficacy of parents and enhance their intention to vaccinate their daughters.

## Data availability statement

The original contributions presented in the study are included in the article/Supplementary material, further inquiries can be directed to the corresponding author.

## Ethics statement

Ethical review and approval was not required for the study on human participants in accordance with the local legislation and institutional requirements. The patients/participants provided their written informed consent to participate in this study.

## Author contributions

LZ was responsible for the theoretical conceptualization and research design of the whole study, designed questionnaire, and sampling method. WK and LZ contacted the online survey platform, data collection, organized the database, and completed the data cleaning. JY performed the statistical analysis and contributed to the review and editing, in particular Sections Methods and Results. JY and LZ provided the financial support. LZ and YC wrote the original manuscript and completed the visualization. All authors have read and agreed to the published version of the manuscript.
