# Supplementary material for: Sociocultural–psychological predictors influencing parents' decision-making regarding HPV vaccination for their adolescent daughters in mainland China: An extended TPB model
Source: Front Public Health. 2023 Jan 6;10:1035658. doi: 10.3389/fpubh.2022.1035658 (PMC9853056; doi:10.3389/fpubh.2022.1035658)
Supplement: Supplementary file 1 [file Table_1.pdf]

Appendix

Table 1. Seven-point Likert scales for 11 constructs were showed as below.

Constructs	Measurement Items	Literature
Perceived Infection Risk (PIR)	PIR1: How worried are you about your daughter being exposed to HPV at present?<br>PIR2: How likely do you think your daughter is to be affected by HPV in the future?<br>PIR3: How seriously do you think that your daughter might become sick from HPV?	van der Linden, S. (1,2);<br>Sarah Dryhurst, et al. (3);<br>Ran Wei et al. (4)
Perceived Vaccine Effectiveness (PVE)	PVE1: I believe that the HPV vaccine is effective in preventing genital HPV infection.<br>PVE2: I believe that the HPV vaccine works to prevent genital HPV infection.<br>PVE3: I believe that if my daughter gets the HPV vaccine, she will be less likely to get genital HPV infection.	Loretta Brabin, et al. (5);<br>Xiaoli Nan and Kelly Madden (6)
Perceived Vaccine Side-Effects	PVSE1: I worry about the short-term side-effects of the HPV	

(PVSE)	vaccine.<br>PVSE2: I worry that the HPV vaccine might negatively affect my daughter's health.<br>PVSE3: I worry that the HPV vaccine might have unknown long-term side-effects.	
Attitude toward Vaccination (AV)	AV1: For my daughter, getting HPV vaccination at 13–15 years of age is beneficial.<br>AV2: For my daughter, getting HPV vaccination at 13–15 years of age is necessary.<br>AV3: For my daughter, getting HPV vaccination at 13–15 years of age is wise.<br>AV4: For my daughter, getting HPV vaccination at 13–15 years of age is important.	Icek Ajzen (7);<br>Abhyankar, Connor, and Lawton (8);<br>Vinita Agarwal (9)
Anticipated Regret (AR)	If I don't get my daughter vaccinated against HPV at 13–15 years of age,<br>AR1: I will feel regret.<br>AR2: I will be troubled.	Conner et al. (10)

	AR3: I will feel guilty.<br>AR4: I will feel disappointed.	
Anticipated Pride (AP)	If I get my daughter vaccinated against HPV at 13–15 years of age,<br>AP1: I will be proud.<br>AP2: I will be happy.<br>AP3: I will be satisfied.	
Descriptive Norms (DN)	DN1: Most parents on the Internet have vaccinated their 13–15-year-old daughters against HPV.<br>DN2: Most girls aged 13–15 years on the Internet are vaccinated against HPV.<br>DN3: Most of my friends have vaccinated their 13–15-year-old daughters against HPV.<br>DN4: Most of my friends' daughters were vaccinated against HPV at 13–15 years of age.<br>DN5: The parents of most of my daughter's classmates have vaccinated their daughters against HPV.	Icek Ajzen (7);<br>Mark Conner and Paul Norman (11)

	DN6: Most of my daughter’s classmates are vaccinated against HPV.	
Injunctive Norms on the Internet (INI)	INI1: People online think that I should vaccinate my 13–15-year-old daughter.<br>INI2: People online approve of me vaccinating my 13–15-year-old daughter against HPV.<br>INI3: People online hope that I vaccinate my 13–15-year-old daughter against HPV.	
Perceived Moral Obligation (PMO)	PMO1: It is my responsibility to get my daughter vaccinated against HPV at 13–15 years of age.<br>PMO2: It is moral to have my daughter vaccinated against HPV at 13–15 years of age.<br>PMO3: It is in my values to have my daughter vaccinated against HPV at 13–15 years of age.<br>PMO4: I would feel incompetent if I didn’t get my daughter vaccinated against HPV at 13–15 years of age.	E. Dubé et al. (12); Chu and Chiu (13)

Self-Efficacy for Vaccination (SEV)	SEV1: I'm confident that I can take my daughter to get vaccinated against HPV.<br>SEV2: It is very easy for my daughter to get the HPV vaccine.<br>SEV3: I can afford to get my daughter vaccinated against HPV.	Mark Conner and Paul Noman (11); Z. Janet Yang (14)
Vaccination Intention (VI)	VI1: How likely would you be to accept vaccination of your daughter with the HPV vaccine sometime soon?<br>VI2: If you were faced with the decision of getting your daughter vaccinated against HPV today, how likely is it that you would choose to accept the vaccine?<br>VI3: How likely would you be to accept your daughter's vaccination against HPV in the future?	Xiaoli Nan and Kelly Madden (6); Rothman et al. (15)

References

1. van der Linden, S. The Social-Psychological Determinants of Climate Change Risk Perceptions:Towards a Comprehensive Model. *Journal of Environmental Psychology.* (2015)41: 112-24. doi: 10.1016/j.jenvp.2014.11.012.

2. van der Linden, S. Determinants and Measurement of Climate Change Risk Perception, Worry, and Concern. In: M. Nisbet, Editor. *Oxford Research Encyclopedia of Climate Science*. Oxford, UK: Oxford University Press (2017). p.1-49.

3. Sarah Dryhurst, Claudia R. Schneider, John Kerr, Alexandra L. J. Freeman, Gabriel Recchia, Anne Marthe van der Bles, David Spiegelhalter & Sander van der Linden. Risk perceptions of COVID-19 around the world. *Journal of Risk Research.* (2020) 23: 994-1006. doi: 10.1080/13669877.2020.1758193.

4. Wei, R., Lo, V.-H., & Lu, H.-Y. Reconsidering the Relationship Between the Third-Person Perception and Optimistic Bias. *Communication Research.* (2007) 34: 665–84. doi: https://doi.org/10.1177/0093650207307903.

5. Loretta Brabin, Stephen A. Roberts, Farah Farzaneh, Henry C. Kitchener. Future acceptance of adolescent human papillomavirus vaccination: A survey of parental attitudes. *Vaccine.* (2006)24: 3087-94. doi: https://doi.org/10.1016/j.vaccine.2006.01.048.

6. Xiaoli Nan & Kelly Madden. HPV Vaccine Information in the Blogosphere: How Positive and Negative Blogs Influence Vaccine-Related Risk Perceptions, Attitudes, and Behavioral Intentions. *Health Communication.* (2012) 27:829-36. doi: 10.1080/10410236.2012.661348.

7. Icek Ajzen. Constructing a TPB Questionnaire: Conceptual and Methodological Considerations. 2006. Available online at: https://citeseerx.ist.psu.edu/viewdoc/download?doi=10.1.1.601.956&rep=rep1&type=pdf

(accessed Aug 10, 2022).

8. Purva Abhyankar, Daryl B. O'connor & Rebecca Lawton. The role of message framing in promoting MMR vaccination: Evidence of a loss-frame advantage. *Psychology, Health & Medicine.* (2008) 13:1-16. doi: 10.1080/13548500701235732.

9. Vinita Agarwal. A/H1N1 Vaccine Intentions in College Students: An Application of the Theory of Planned Behavior. *Journal of American College Health.* (2014) 62:416-24. doi: 10.1080/07448481.2014.917650.

10. Conner, M., Godin, G., Sheeran, P., & Germain, M. Some feelings are more important: Cognitive attitudes, affective attitudes, anticipated affect, and blood donation. *Health Psychology.* (2013) 32: 264-72. doi: https://doi.org/10.1037/a0028500.

11. Mark Conner and Paul Norman. *Predicting Health Behaviour: Research and Practice with Social Cognition Models.* Buckingham: Open University Press (1996).

12. PIN-YU CHU & JA-FUN CHIU. Factors Influencing Household Waste Recycling Behavior: Test of an Integrated Model. *Journal of Applied Social Psychology.* (2003)33: 604-26. doi: https://doi.org/10.1111/j.1559-1816.2003.tb01915.x.

13. Z. Janet Yang. Predicting Young Adults' Intentions to Get the H1N1 Vaccine: An Integrated Model. *Journal of Health Communication.* (2015)20: 69-79. doi: 10.1080/10810730.2014.904023.

14. Dubé E, Gagnon D, MacDonald N, Bocquier A, Peretti-Watel P, Verger P. Underlying factors impacting vaccine hesitancy in high income countries: a review of qualitative studies. *Expert Rev Vacc.* (2018) 17:989-1004. doi: https://doi.org/10.1080/14760584.2018.1541406.

15. Rothman AJ, Martino SC, Bedell BT, Detweiler JB, Salovey P. The Systematic Influence

of Gain-and Loss-Framed Messages on Interest in and Use of Different Types of Health Behavior. Personality and Social Psychology Bulletin. (1999) 25:1355-69. doi:10.1177/0146167299259003.
